# Direct from the COVID-19 crisis: research and innovation sparks in Brazil

**DOI:** 10.1186/s12961-020-00674-x

**Published:** 2021-01-21

**Authors:** Mário Fabrício Fleury Rosa, Everton Nunes da Silva, Christina Pacheco, Marcos Vinícius Pereira Diógenes, Christopher Millett, Carlos Augusto Grabois Gadelha, Leonor Maria Pacheco Santos

**Affiliations:** 1 Universidade de Brasília (UnB), Brasília, DF Brazil; 2 Universidade Estadual do Rio Grande do Norte (UERN), Mossoró, RN Brazil; 3 Imperial College London, London, England United Kingdom; 4 Universidade de São Paulo (USP), São Paulo, Brazil; 5 Fundação Oswaldo Cruz (Fiocruz), Rio de Janeiro, Brazil

**Keywords:** COVID-19, Brazil, Research innovation and development, Health economic-industrial complex

## Abstract

**Background:**

The coronavirus disease 2019 (COVID-19) pandemic has spread throughout more than 160 countries, infecting millions of people worldwide. To address this health emergency, countries have organized the flow of production and innovation to reduce the impact on health. This article shows the response of the Brazilian scientific community to meet the urgent needs of the public unified health system [SUS], aiming to guarantee universal access to an estimated population of 211 million. By December 2020, Brazil had recorded more than six million cases and approximately 175,000 deaths.

**Methods:**

We collected data on research, development and innovation projects carried out by 114 public universities (plus Oswaldo Cruz Foundation [Fiocruz] and Butantan Institute), as reported on their websites. Additionally, we examined the studies on COVID-19 approved by the National Comission for Research Ethics, as well as those reported on the Ministry of Education website as of May 15, 2020.

**Results:**

The 789 identified projects were classified according to research categories as follows: development and innovation (*n* = 280), other types of projects (*n* = 226), epidemiologic research (*n* = 211), and basic research on disease mechanisms (*n* = 72). Most proposals focused on the development and innovation of personal protective equipment, medical devices, diagnostic tests, medicines and vaccines, which were rapidly identified as research priorities by the scientific community. Some promising results have been observed from phase III vaccine trials, one of which is conducted in partnership with Oxford University and another of which is performed with Sinovac Biotech. Both trials involve thousands of volunteers in their Brazilian arms and include technology transfer agreements with Fiocruz and the Butantan Institute, respectively. These vaccines proved to be safe and effective and were immediately licensed for emergency use. The provision of doses for the public health system, and vaccination, started on January 17, 2021.

**Conclusions:**

The mobilized Brazilian scientific community has generated comprehensive research, development and innovation proposals to meet the most urgent needs. It is important to emphasize that this response was only possible due to decades of investment in research, development and innovation in Brazil. We need to reinforce and protect the Brazilian science, technology and innovation system from austerity policies that disregard health and knowledge as crucial investments for Brazilian society, in line with the constitutional right of universal health access and universal health coverage.

## Background

The coronavirus disease 2019 (COVID-19) pandemic has forced health authorities around the world to propose social distancing and other measures, mainly due to the ease of transmission and dissemination of the virus through the air [1]. In addition to personal protective equipment (PPE) for populations and health professionals, products such as mechanical respirators have become essential to combat the deadly impact of the pandemic. The disease has spread to more than 160 countries, infecting millions of people globally. Estimates of severe cases have been the main concern of health authorities worldwide [2].

This global health emergency has demanded urgent responses to lessen the impact of the pandemic, forcing the international scientific community to develop and improve interventions that can assist public policies in slowing the spread of the pandemic in their communities. In Brazil, the research, development and innovation (RD&I) sectors have suffered severe budget cuts since 2018 [3]. Recently, government officials have denied scientific findings by saying “So what!” as described in *The Lancet* editorial, “COVID-19 in Brazil: So What?” [4]. However, universities and public research centers have remained focused on their social responsibility and have continued to work diligently to help control and mitigate the COVID-19 pandemic, using a voluntary work force and the existing research infrastructure in a clear demonstration of strong institutional resilience.

On February 26, 2020, the first case of COVID-19 was identified in Brazil. By May 15, when we conducted this survey, 218,000 confirmed cases and 14,000 deaths due to COVID-19 had been reported. These figures had jumped to six million cases and 175,000 deaths by December 2020; with regard to the number of cases and deaths, Brazil ranks third internationally. This illustrates the challenge facing our public Unified Health System (SUS) in providing universal access to care and universal health coverage, as recommended by the Pan American Health Organization (PAHO) [5] and guaranteed by the Brazilian Constitution, to an estimated population of 211 million in a country with a vast territory and wide regional and social inequalities [6].

Given this context, this article investigates how the Brazilian scientific community responded during the very early stages of the COVID-19 epidemic.

### The research and development (R&D) scenario in Brazil

The Brazilian science and technology (S&T) system is composed of public universities and public research centers; this helps to explain how health research has developed in Brazil over time. Research in the area of COVID-19 shows that even with the scarcity of funding, universities and public research centers support national scientific development.

In Brazil, scientific research in the field of public health in the early twentieth century encouraged the development of the national health research systems [7], starting with the creation of public research centers such as the Oswaldo Cruz Foundation (Fiocruz) in Rio de Janeiro in 1900 and the Butantan Institute in São Paulo in 1901. The creation of the Brazilian Academy of Sciences (ABC) followed shortly thereafter, in 1916. The history of Brazil’s health research system is intertwined with the creation of a network of publicly funded universities. The first public university was Amazonas Federal University, founded in 1909, followed by substantial federal government investments that led to the creation of 36 universities by 1974. After three decades of stagnation and deterioration (1975–2004), investments in higher education accelerated substantially under Presidents Lula and Dilma (2003–2016). They launched the “Restructuring and Expansion of Federal Universities Project,” REUNI (2003–2012), which created 15 new universities, in addition to refurbishing installations, infusing the faculty with qualified researchers, increasing available spaces for students in undergraduate courses, expanding the availability of evening courses and promoting pedagogical innovations, all with the aim of reducing social inequalities in the country [8]. In fact, 23% of the existing federal universities started during this period, resulting in an unprecedented democratization of access to high-level education. At present, this network of public universities encompasses 68 highly qualified and autonomous universities that are completely free (unlike universities in the United States and European countries) and offer scholarships (tax-free) for undergraduate and graduate (masters, doctoral) students as well as postdocs. In addition, there are 41 public universities maintained by state governments and five financed by municipal governments. This network of 114 institutions [9] is undoubtedly an important and integral part of the scientific and cultural heritage of the nation [10].

The implementation of the national science and technology policy started in 1951, with the creation of the National Council for Scientific and Technological Development (CNPq) and the Coordination for the Improvement of Higher Education Personnel (CAPES), which contributed to transforming universities and public research centers into vectors for RD&I. From 2004 to 2014, the coordinated efforts of the Ministry of Health’s Department of Science and Technology (DECIT), CNPq and CAPES encouraged the establishment of a health science, technology and innovation policy [11]. In addition, an agenda of health research priorities was generated and implemented, with 3586 projects financed [12]. Starting in 2004, federal RD&I investments were also on the rise. A recent report from the Brazilian Institute of Applied Economic Research (IPEA) analyzed RD&I expenditures from 2000 to 2020. Prior to 2004, the expenditures amounted to approximately R$4 million per year. This amount increased steadily, reaching R$13 million in 2015, but afterwards sharp reductions occurred, and by 2018 the budget had been reduced to R$5.1 million [3].

Brazil ranked 13th in international scientific publications in 2015 [11]. The development of the public university research infrastructure, as mentioned before, together with federal RD&I investments from 2004 to 2015 were decisive factors facilitating the achievement of this rank [3].

### Development, innovation and the health economic-industrial complex in Brazil

Because of the COVID-19 public health emergency, societies in virtually all countries had to organize their flow of production and innovation in the hope of reducing the impact on the health systems and guaranteeing adequate health care for the population. The RD&I sectors in each country have assumed the immense responsibility of generating the domestic responses to reduce the impacts on the healthcare and economic systems of their regions because, given the magnitude of this pandemic, imports of healthcare products and supplies are limited by the scarcity of products on the international market. Those countries with established health production systems (that involve different production chains) are more likely to avoid shortages of raw materials and finished products that are essential for combatting the pandemic.

However, in Brazil, the knowledge generated by ongoing research that led to Brazil ranking 13th in terms of scientific publications did not lead to proportional advances in the development of organizational processes and technologies. This could explain why, in 2016, Brazil ranked 69th on the Global Innovation Index. During the pandemic, Brazilian universities struggled to overcome the wide disparity between the high level of production of scientific knowledge and the low level of scientific innovation. In the “ecosystem” of economic and social development in which the production sector and government participate, the university sector must play a fundamental role in transforming knowledge into solutions that confer benefits to society [13].

From 2004 to 2015, a concerted effort was made in Brazil to stimulate the triple helix model of innovation, which describes the interactions among universities, industries and governments [14]. Such interactions are considered key to innovation in increasingly knowledge-based societies, such as China, as well as in other developing countries [15]. The paradigm known as the Brazilian health economic-industrial complex (HEIC) considers the health sector to be part of the production and innovation system that generates wealth and jobs; this concept was present in the advancement of SUS in recent decades, during which it operated in accordance with the market. The rationale that guided the public policies resulting from this paradigm emphasized the systemic approach and the use of states’ purchasing power to push sectorial development [16]. From this point of view, the health sector is part of the developmental agenda and supports innovation and economic development through health industrial complexes [17].

Given this context, this article investigates how the Brazilian scientific community has responded to the threat of COVID-19. The knowledge accrued by universities, institutes and public health research centers was challenged by the COVID-19 pandemic, and the scientific sector responded immediately by producing health solutions to mitigate the destructive progress of the ongoing pandemic. We intend to show the immediate response and commitment of the Brazilian scientific community. Most of these actions were taken as the result of the scientists’ own initiative and desire to meet the most urgent needs of the population affected by COVID-19, and not in response to specific calls for proposals.

## Methods

To analyze the actions taken by Brazilian scientists to meet the needs arising from the COVID-19 pandemic, we collected official RD&I projects carried out by 114 Brazilian public universities (plus Fiocruz and Butantan), as reported on their institutional websites (access May 10–15, 2020). Additionally, we examined all the studies on COVID-19 that had been submitted to and approved by the National Comission for Research Ethics (CONEP) as of May 15, 2020 [18]. Third, we analyzed all the RD&I proposals available on the Ministry of Education official website on May 15, 2020, which were obtained with web-scraping computational tools.

We then analyzed and classified the projects into categories according to information obtained from the proposals’ titles and/or summaries. The following categories emerged:

Basic research on disease mechanisms: genetic sequencing, viral mutations, physiopathology, immunological profiles and clinical manifestations of COVID-19.

Epidemiologic research: distribution and evolution of the disease, risk factors for COVID-19.

Development and innovation (D&I), organized into five subcategories: (a) personal protective equipment (PPE): face shields, masks, disinfection mechanisms; (b) medical devices: respirators, ventilators, mobile ICUs; (c) diagnostic tests: novel tests, rapid tests, alternative body fluid samples, test accuracy; (d) vaccines: development of vaccines against severe acute respiratory syndrome coronavirus 2 (SARS-CoV-2); (e) medications and therapy: drugs, novel treatments for COVID-19.

Other types of projects: research relevant to COVID-19 and its social, psychological and economic consequences.

The projects under the category of D&I demonstrate the potential for developing new technologies, resulting in the transfer of technology from universities to the production sector and interactions with the health economic-industrial complex.

## Results and discussion

There was a rapid reaction from Brazilian scientists; the search on the official websites of each of the 114 public universities, which are distributed throughout Brazil, retrieved information on 551 R&D projects, the majority of which originated in the Southeast region. As of May 15, 2020, scientists had presented 270 research protocols related to COVID-19 to CONEP, including 46 clinical trials and 224 observational studies, which were located in 24 of the 27 states. Finally, the Ministry of Education's official website reported approximately 73 proposals. In total, 894 initiatives were retrieved; after the elimination of duplicates, 789 RD&I projects were analyzed and are shown in the following tables/figures. The details are provided in Additional files 1 (Creation of Public Federal Universities and Public State Universities in Brazil, 1909–2018), 2 (Federal investments in research and development. Brazil 2000–2020) and 3 (Research, development & innovation about COVID-19 by institution, Brazil 2020).

Table 1 presents the research projects, classified into categories as described before. With regard to the four broad categories, the majority of proposals (*n* = 280) were D&I projects that focused on PPE, medical devices, diagnostic tests, vaccines and medicines, which were rapidly identified as research priorities by the scientific community. These topics are extremely important under the current circumstances for the development of the health economic-industrial complex and the Brazilian capacity to attend to health needs [16].

**Table 1:** Research, development and innovation projects focusing on COVID-19 led by public universities and public health research institutes

Research category (number of projects per subcategory)	Number	Proportion (%)
Development and innovation (D&I)	280	35.6
Personal protective equipment (*n* = 45)		
Medical devices (*n* = 36)		
Diagnostic tests (*n* = 89)		
Vaccines (*n* = 10)		
Medications and therapy (*n* = 100)		
Other types of COVID-19 research projects	226	28.6
Epidemiologic research	211	26.7
Basic research (focusing on disease mechanisms)	72	9.1
Total	789	100.0

Brazil, 2020: Additional file with detailed descriptions available

The initiatives indicate a focus on translational health research [19] involving the transfer of knowledge generated in the basic sciences to the production of new products such as medicines, equipment, PPE, diagnostic tests and innovative treatment options. These 280 projects aim to bridge the gap between bench research and its application in health [19, 20].

**Fig. 1:**
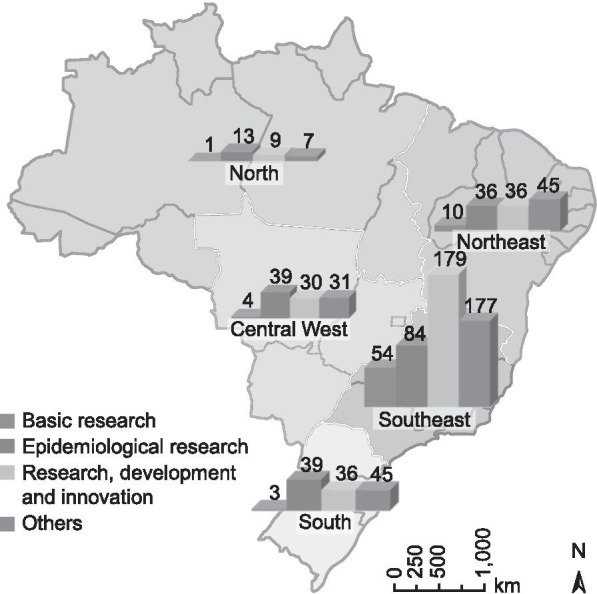
Regional distribution of research on COVID-19 in Brazil, May 15, 2020

The map of the Brazilian geographic regions in Fig. 1 shows the distribution of research proposals developed by public universities, stratified by the project categories. Public universities from all Brazilian regions were involved. The Southeast and Northeast regions accounted for 55.0% and 16.1% of the proposals, respectively. The Central West, South and North regions accounted for 13.2%, 11.9% and 3.8%, respectively.

In terms of financial support, there was information on 140 projects that received funding, 116 of which were developed by federal universities and 24 led by state universities. The project category most likely to receive funds was D&I (25.7%), especially vaccine development (50.0%), as such projects present the health solutions most in line with the urgent needs of the healthcare system (Table 2). At the time of our data collection, the number of research proposals that had received funding was relatively low. This first analysis showed that despite inadequate funding, the Brazilian D&I sector offered scientific and technological options capable of strengthening the health production chain in response to the public health emergency caused by the spread of COVID-19.

**Table 2:** Research, development and innovation projects with financial support focusing on COVID-19 led by public universities and public research institutes

Research category (% of financed projects per subcategory)	Number of projects	Number of financed projects	Proportion of financed projects (%)
Research, development and innovation	280	72	25.7
Personal protective equipment (26.7%)			
Medical devices (38.9%)			
Diagnostic tests (15.7%)			
Vaccines (50.0%)			
Medications and therapy (27.9%)			
Other types of COVID-19 research projects	226	13	5.8
Epidemiologic research	211	40	19.0
Basic research (focusing on disease mechanisms)	72	15	20.8
Total	789	140	17.7

Brazil, 2020: Additional file with detailed descriptions available

Out of the 789 research projects included and analyzed, there were only 10 vaccine projects (50% of which were financed). Considering the highly sophisticated laboratory facilities and clinical requirements for vaccine development, this is reasonable. However, there is evidence that the most promising vaccines for SUS and for the Brazilian population may come from two of these vaccine trials. One candidate vaccine based on the spike (S) glycoprotein is developed in partnership with Oxford University in the United Kingdom. The phase III study is under way and plans to enroll more than 30,000 volunteers worldwide. Brazil will participate in the tests, providing approximately 2000 volunteers under the supervision of the Federal University of São Paulo (UNIFESP) [10] and Fiocruz [21]. This partnership involves the production, by Fiocruz, of 100 million doses of the vaccine for the Brazilian population; 30 million doses could be available in December 2020/January 2021 [21]. The other trial was of the CoronaVac vaccine, developed by the Chinese pharmaceutical company Sinovac Biotech, which involved 9000 volunteers in São Paulo, Brazil, in the final phase III trial. If proven effective and safe, national production will commence immediately due to technology transfer agreements with Butantan in São Paulo. The provision of doses to the SUS will be possible as early as June 2021 [22].

There are 211 projects classified as epidemiologic research, which include the development of several observatories, surveillance systems and mobile phone apps for COVID-19 monitoring at the local and national levels, especially for vulnerable populations. One example is MONITORA COVID-19, developed by Fiocruz (https://bigdata-covid19.icict.fiocruz.br/), and another is the COVID-19 BR Observatory, established by the Federal University of ABC (https://covid19br.github.io/).

The COVID-19 health emergency is reminding all members of the scientific community of the reasons we embarked on research careers: we know that research is vital and valuable and can save lives. During this crisis, we are accelerating research production [23]. It is reassuring that society has come to recognize the fundamental role of solid scientific evidence [24].

## Conclusions

Strong mobilization of the Brazilian scientific community took place in a very short time to respond to the COVID-19 crisis, presenting 789 scientific proposals to address the most urgent problems posed by the pandemic. The interaction of universities, industries and governments is essential. In the absence of this linkage in Brazil, it is virtually impossible to perform translational research, that is, to take the results from the bench to the bedside, and from there to the healthcare system. It is necessary to enhance and encourage the interaction between universities and public research centers, which are involved in the production of scientific knowledge, and private companies, which specialize in production on an industrial scale.

However, it must be emphasized that the rapid response observed in this survey was only possible due to decades of investment in the RD&I system in Brazil. We need to reinforce and protect this system from austerity policies that disregard health and knowledge as crucial investments for Brazilian society, in line with the constitutional right to universal health access and universal health coverage.

## Supplementary Information

**Additional file 1**: Creation of Public Federal Universities and Public State Universities in Brazil 1909–2018.

**Additional file 2**: Federal investments in research and development in Brazil 2000–2020.

**Additional file 3**: Research, development & innovation about COVID-19 by institution, Brazil 2020.

## Data Availability

All data generated or analyzed during this study are included in this published article and its Additional information files.

## Abbreviations

PPE: Personal protective equipment
SUS: Unified Health System
PAHO: Pan American Health Organization
Fiocruz: Oswaldo Cruz Foundation
ABC: Brazilian Academy of Sciences
REUNI: Restructuring and Expansion of Federal Universities Project
CNPq: National Council for Scientific and Technological Development
CAPES: Coordination for the Improvement of Higher Education Personnel
RD&I: Research, development and innovation
DECIT: Ministry of Health’s S&T Department
IPEA: Institute of Applied Economic Research
HEIC: Brazilian health economic-industrial complex
UNIFESP: Federal University of São Paulo
D&I: Development and innovation

## References

[1] Yang J, Zheng Y, Gou X, Pu K, Chen Z, Guo Q (2020). Prevalence of comorbidities and its effects in patients infected with SARS-CoV-2: a systematic review and meta-analysis. Int J Infect Dis.

[2] OPAS. Organização Pan-Americana de Saúde. Folha informativa COVID-19 - Escritório da OPAS e da OMS no Brasil. 2020. https://www.paho.org/bra/index.php?option=com_content&view=article&id=6101:covid19&Itemid=875. Accessed 9 May 2020.

[3] Institute of Applied Economic Research (IPEA). Federal investments in research and development: estimates for the period 2000–2020. Brasília: IPEA; 2020.

[4] The Lancet Editorial. COVID-19 in Brazil: “So what?” 2020;395(10235):P1461. 10.1016/S0140-6736(20)31095-3.

[5] OPAS. Organização Pan-Americana de Saúde. Strategy for Universal Access to Health and Universal Health Coverage. Washington, D.C., USA. 2014. https://iris.paho.org/handle/10665.2/28276. Accessed 31 May 2020.

[6] Paim J, Travassos C, Almeida C, Bahia L, Macinko J (2011). The Brazilian health system: history, advances and challenges. Lancet.

[7] Pang T, Sadana R, Hanney S, Bhutta Z, Hyder A, Simon J. Knowledge for better health: a conceptual framework and foundation for health research systems. Bull World Health Organ. 2003;81(11):815–820. https://apps.who.int/iris/handle/10665/72104 PMC2572351 14758408

[8] REUNI. Reestruturação e expansão das universidades federais. O que é o REUNI. 2010. http://reuni.mec.gov.br/o-que-e-o-reuni. Accessed 12 May 2020.

[9] e-MEC. Cadastro Nacional de Cursos e Instituições de Educação Superior Cadastro e-MEC. 2017. http://emec.mec.gov.br/ Accessed 9 May 2020.

[10] Agência Brasil. Brasil pode ter prioridade no uso da vacina de Oxford contra covid-19. 2020. https://agenciabrasil.ebc.com.br/saude/noticia/2020-06/brasil-pode-ter-prioridade-no-uso-da-vacina-de-oxford-diz-reitora. Accessed 5 June 2020.

[11] Guimarães R, Santos LMP, Angulo-Tuesta A, Serruya SJ (2006). Defining and implementing a national policy for science, technology, and innovation in health: lessons from the Brazilian experience. Cad Saude Publica..

[12] Santos LMP, Moura EC, Barata RDCB, Serruya SJ, da Motta ML, Elias FTS (2011). Fulfillment of the Brazilian agenda of priorities in health research. Health Res Policy Syst.

[13] Fernandes ACS (2017). Brazilian social and economic development depends on science. Braz J Med Hum Health.

[14] Rosa MFF, Guimarães SMF, Dominguez AGD, Assis RS, Reis CB, Rosa SDSRF (2019). Development of hard technology for the treatment of diabetic foot: a case study from the perspective of collective health. Saúde Debate..

[15] Li R, Weihua F (2019). University-industry-government relations of the Ministry of Industry and Information Technology (MIIT) universities: the perspective of mutual information. PLoS ONE.

[16] Gadelha CAG, Temporão JG (2018). Development, innovation and health: the theoretical and political perspective of the Health Economic-Industrial Complex. Ciênc Saúde Coletiva.

[17] O Globo. ‘Não podemos ter um SUS com tamanha dependência’, diz pesquisador da Fiocruz em meio à crise do coronavírus. 2020. https://oglobo.globo.com/sociedade/coronavirus/nao-podemos-ter-um-sus-com-tamanha-dependencia-diz-pesquisador-da-fiocruz-em-meio-crise-do-coronavirus-24366231. Accessed 13 May 2020.

[18] Conselho Nacional de Saúde. Comissão Nacional de Ética em Pesquisa, Research ethics bulletin—special edition 16. 2020. https://conselho.saude.gov.br/publicacoes-conep. Accessed 19 May 2020.

[19] Barreto JOM, Silva EN, Gonçalves RG, Rosa SSRF, Felipe MSS, Santos LMP (2019). Translational research in public health: challenges of an evolving field. Saude Debate.

[20] Pacheco C, Ceccatto VM, Maia CM, Rosa SSRF, Leite CRM (2019). Translational research in the post-genomic era: advances in the field of transcriptomics. Saude Debate.

[21] Correio Braziliense. Brasil anuncia parceria com Oxford para produzir vacina contra coronavírus, 2020. https://www.correiobraziliense.com.br/app/noticia/brasil/2020/06/27/interna-brasil,867407/brasil-anuncia-parceria-com-oxford-para-produzir-vacina-contra-coronav.shtml. Accessed 27 June 2020.

[22] Instituto Butantan. Butantan e Governo de SP vão testar e produzir vacina inédita contra coronavírus. 2020. http://www.butantan.gov.br/noticias/butantan-e-governo-de-sp-vao-testar-e-produzir-vacina-inedita-contra-coronavirus. Accessed 05 June 2020.

[23] Turner T, El-Jardali F (2020). The crucible of COVID-19: what the pandemic is teaching us about health research systems. Health Res Policy Syst.

[24] Folha de São Paulo. Na contramão do governo, brasileiros acreditam mais na ciência. 2020. https://piaui.folha.uol.com.br/na-contramao-do-governo-brasileiros-acreditam-mais-na-ciencia/. Accessed: 26 May 2020.

