# Supplementary figures and images for: Direct from the COVID-19 crisis: research and innovation sparks in Brazil
Source: Health Res Policy Syst. 2021 Jan 21;19:10. doi: 10.1186/s12961-020-00674-x (PMC7819618; doi:10.1186/s12961-020-00674-x)

Federal

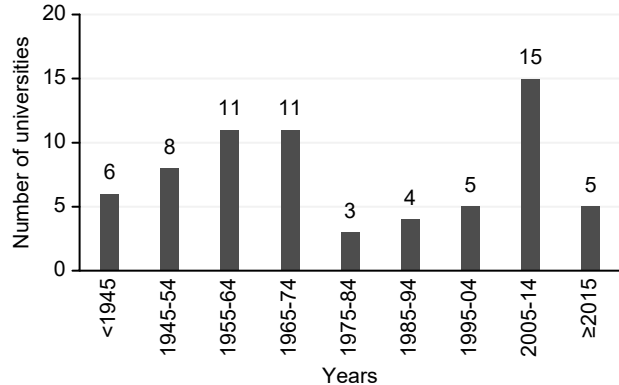

State

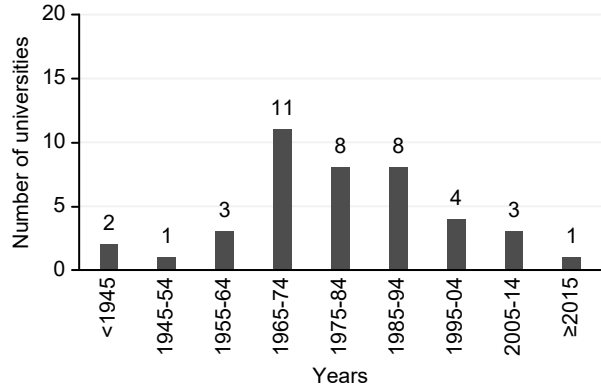

Supplement: Supplementary file 1 — Additional file 1: Creation of Public Federal Universities and Public State Universities in Brazil 1909–2018. [file 12961_2020_674_MOESM1_ESM.pdf]

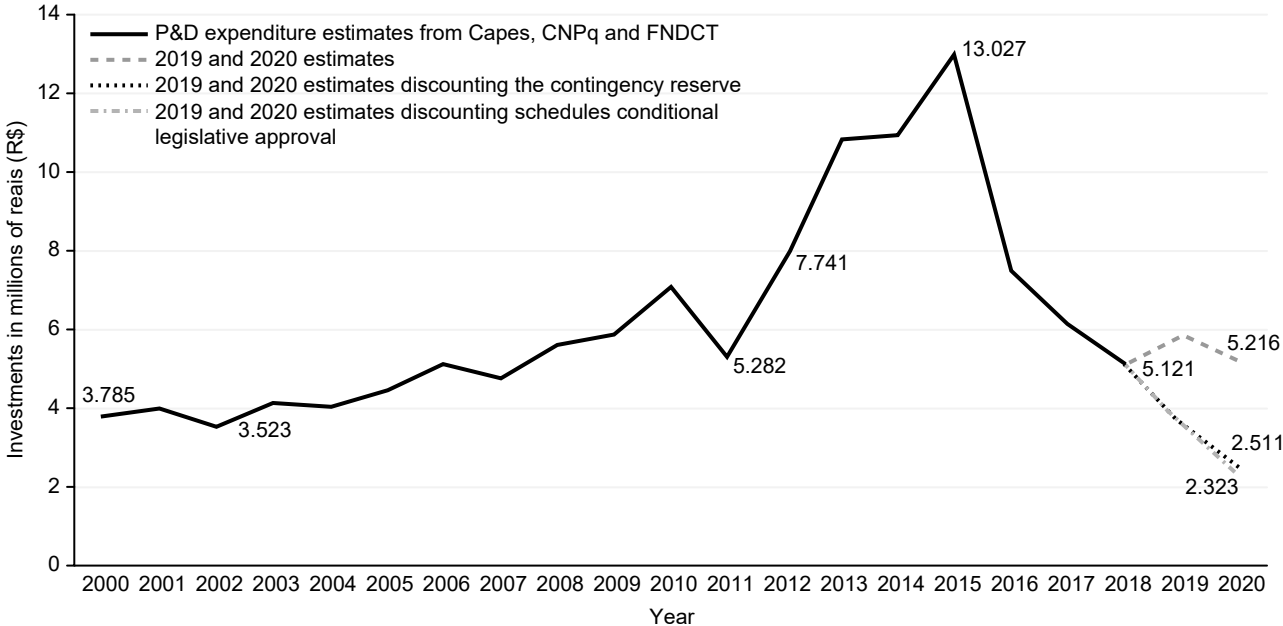

Supplement: Supplementary file 2 — Additional file 2: Federal investments in research and development in Brazil 2000–2020. [file 12961_2020_674_MOESM2_ESM.pdf]
